# The role of the fat mass and obesity associated gene (*FTO*) in breast cancer risk

**DOI:** 10.1186/1471-2350-12-52

**Published:** 2011-04-13

**Authors:** Virginia Kaklamani, Nengjun Yi, Maureen Sadim, Kalliopi Siziopikou, Kui Zhang, Yanfei Xu, Sarah Tofilon, Surbhi Agarwal, Boris Pasche, Christos Mantzoros

**Affiliations:** 1 Cancer Genetics Program, Division of Hematology/Oncology, Department of Medicine and Robert H. Lurie Comprehensive Cancer Center, Feinberg School of Medicine, Northwestern University, 676 N St Clair st suite 850, Chicago, IL 60611,USA; 2 Section on Statistical Genetics, Department of Biostatistics, School of Public Health, University of Alabama at Birmingham, 1665 University Blvd, Ryals Bldg. 317F, Birmingham, AL 35294, USA; 3 Cancer Genetics Program, Division of Hematology/Oncology, Department of Medicine and Robert H. Lurie Comprehensive Cancer Center, Feinberg School of Medicine, Northwestern University, 303 E Superior Street, Chicago, IL60611, USA; 4 Department of Pathology, Northwestern Memorial Hospital, 675 N St Clair st, Chicago, IL 60611, USA; 5 Section on Statistical Genetics, Department of Biostatistics, School of Public Health, University of Alabama at Birmingham, 1665 University Blvd, Ryals Bldg. 327H, Birmingham, AL 35294, USA; 6 Division of Hematology/Oncology, Department of Medicine and Robert H. Lurie Comprehensive Cancer Center, Feinberg School of Medicine, Northwestern University, 303 E Superior Street, Chicago, IL 60611, USA; 7 Department of Medicine and Robert H. Lurie Comprehensive Cancer Center, Feinberg School of Medicine, Northwestern University, 303 E Superior Street, Chicago, IL 60611, USA; 8 Division of Hematology/Oncology and Comprehensive Cancer Center, University of Alabama, 1802 6th Avenue South, NP 2566, Birmingham, AL35294, USA; 9 Division of Endocrinology and Metabolism, Department of Medicine, Beth Israel Deaconess Medical Center (BIDMC), Harvard Medical School, 330 Brookline Avenue FD-876, Boston, MA 02215, USA

## Abstract

**Background:**

Obesity has been shown to increase breast cancer risk. *FTO *is a novel gene which has been identified through genome wide association studies (GWAS) to be related to obesity. Our objective was to evaluate tissue expression of FTO in breast and the role of FTO SNPs in predicting breast cancer risk.

**Methods:**

We performed a case-control study of 354 breast cancer cases and 364 controls. This study was conducted at Northwestern University. We examined the role of single nucleotide polymorphisms (SNPs) of intron 1 of *FTO *in breast cancer risk. We genotyped cases and controls for four SNPs: rs7206790, rs8047395, rs9939609 and rs1477196. We also evaluated tissue expression of FTO in normal and malignant breast tissue.

**Results:**

We found that all SNPs were significantly associated with breast cancer risk with rs1477196 showing the strongest association. We showed that FTO is expressed both in normal and malignant breast tissue. We found that *FTO *genotypes provided powerful classifiers to predict breast cancer risk and a model with epistatic interactions further improved the prediction accuracy with a receiver operating characteristic (ROC) curves of 0.68.

**Conclusion:**

In conclusion we have shown a significant expression of FTO in malignant and normal breast tissue and that *FTO *SNPs in intron 1 are significantly associated with breast cancer risk. Furthermore, these *FTO *SNPs are powerful classifiers in predicting breast cancer risk.

## Background

Breast cancer is the most common malignancy in women in developed countries. In 2009 an estimated 194,280 new cases of breast cancer were diagnosed in the US[1]. Several studies have associated obesity and weight gain with breast cancer risk[2,3] and there is evidence that weight loss, as well as, decrease in fat consumption may lead to decreased risk for breast cancer[4,5]. Diabetes mellitus (DM) has also been associated with breast cancer risk [6]. This association between DM, obesity and breast cancer lead us to evaluate the role of genes, which have been found to be associated with diabetes and obesity, in predicting breast cancer risk.

The fat mass and obesity (*FTO*) associated gene was recently found in several genome wide association studies (GWAS) to be associated with obesity and type II DM[7-11]. More specifically single nucleotide polymorphisms (SNPs) in intron 1 of *FTO*, such as rs9939609 have been significantly associated with obesity[7-11]. Although its role remains essentially unclear, it seems that *FTO *encodes a 2-oxoglutarate-dependent nucleic acid demethylase[12]. It is localized in the nucleus and is abundant in the brain[12]. *FTO *has been shown to be expressed in other tissue as well including the pancreas, skeletal muscle and white adipose tissue although its expression in these tissues is significantly lower than its expression in the hypothalamus and the cerebellum[12].

Given the association between obesity and breast cancer [2,3] we elected to perform a case control study to evaluate the role of SNPs in intron 1 of *FTO *in predicting breast cancer risk. We selected SNPs in intron 1 because that was the region that was more strongly associated with obesity [8,13].

## Methods

### Study Participants

Our case-control study included 354 cases of breast cancer and 364 controls from Northwestern University in Chicago, Illinois. Cases and controls were frequency matched for age by 5 years, race, and sex. Race was included as a variable because breast cancer risk varies among various ethnic groups. Consecutive cases with a biopsy-confirmed diagnosis of breast adenocarcinoma were recruited from the medical oncology clinics affiliated with the Northwestern Medical Faculty Foundation from August 1, 2007, through October 28, 2008. Information on the subtype of breast cancer was obtained from the chart review and had been confirmed at Northwestern University. The response rate was 96% and a blood sample was obtained from each recruited patient. All cases signed an informed consent for genetic studies and the protocol was approved by the Institutional Board Review (IRB) of Northwestern University. Controls were chosen randomly from a total of 5578 patients without a diagnosis of cancer at the time of enrollment who were recruited between October 31, 2002, and December 31, 2007. Controls were matched for age within 5 years, sex, and ethnic status. Cases and controls were not matched by chronic disease status. Controls came from the NUgene Project, a biospecimen repository compliant with the Health Insurance Portability and Accountability Act and approved by the IRB, with questionnaire data and longitudinal medical information from participating patients treated at hospitals and outpatient clinics affiliated with Northwestern University. Potential participants are approached either by a genetic counselor or by a physician. The overall response rate has been 25%. The 2 main reasons for refusal to participate were lack of time and concerns about privacy. The repository represents a clinically diverse population with housing samples and data from healthy individuals and patients with the most common health conditions such as diabetes, cancer, and autoimmune and cardiovascular diseases. Participants signed an IRB-approved informed consent to allow distribution and use of de-identified samples and data for a broad range of research. The ability to regularly update participant health status allows NUgene to provide investigators with the most appropriate samples for their research. Controls were selected from NUgene participants without any self-reported history of cancer. Participants with *International Classification of Diseases, Ninth Revision *codes indicating a diagnosis of cancer were excluded.

### DNA Isolation

DNA from whole blood lymphocytes was extracted using the QIAamp DNA Blood Mini Kit (Qiagen, Hilden, Germany) and was stored at -20°C until use for genotyping. The samples were stored at -20°C.

### Selection of SNPs

We selected four SNPs in *FTO *to evaluate. These SNPs were selected based on previous data from GWAS. More specifically rs9939609 has been previously identified to be significantly associated with obesity and DM [8-11]. Furthermore, other SNPs in the same linkage disequilibrium (LD) block (rs1121980, rs9939973, rs7193144, rs9940128, rs8050136) were found to be associated with obesity [13]. The SNPs we selected to evaluate are all in intron 1 of *FTO *and represent LD block 6 (rs7206790 and rs8047395) and LD block 7 (rs9939609 and rs1477196). This region has been suggested to be strongly associated with obesity[8,9,13].

### Genotyping

Genotyping for the four SNPs was performed by Taqman SNP allelic discrimination by means of an ABI 7900HT (Applied Biosystems, Forest City, California). Results were ascertained by using the SDS software version 2.3 (Applied Biosystems). All results were automatically called (ie, the device displays the genotypes automatically with a 95% certainty). A total of 5% of samples were genotyped in duplicate and showed 100% concordance.

### Breast Tissue

Tissue was obtained from the tissue core facility at Northwestern University. A total of 100 samples were received. Of them 50 were normal breast tissue and 50 were breast cancer tissue. Of the breast tumor tissue 25 samples were ER negative and 25 were ER positive, 20 were PR negative and 30 were PR positive, 41 samples were Her2 negative and 7 Her2 positive. We received 30-40 mg of tissue per sample.

### RNA Extraction and cDNA synthesis

Breast Tissues in RNAlater were flash frozen in liquid N_2_. Tissues were crushed into fine powder and were subsequently lysed in 1 ml of TRIzol^® ^Reagent and 200 ul of chloroform for phase separation. Nucleic Acid Solution (Applied Biosystems, Foster City, CA) was added to the clear (RNA) phase in 3:1 ratio and then added equal volume of 70% EtOH. Mixture was passed through the red filters from the Gen Elute Mammalian Total RNA Miniprep Kit (Sigma-Aldrich, St. Louis, MO). The filters were washed by wash buffers 1 and 2 then eluted using TE buffer. cDNA synthesis from RNA was performed using the TaqMan^® ^Reverse Transcription Reagents (Applied Biosystems, Foster City, CA). cDNA was amplified using TaqMan^® ^PreAmp Master Mix (Applied Biosystems, Foster City, CA). RNA was successfully extracted from all but one sample.

### RT-PCR

The transcription levels for FTO and GAPDH were quantified using the ABI 7900HT Real Time PCR System (Applied Biosystems, Foster City, CA). Primers and probes were designed by Applied Biosystems. Samples are heated at 50°C for 2 min, 95°C for 10 min, followed by 40 cycles of 95°C for 15 sec, and 60°C for 1 min using Taqman^® ^Gene Expression Master Mix (Applied Biosystems, Foster City, CA). Results were normalized to the levels of GAPDH (Taqman^® ^probes, Applied Biosystems) and relative quantification using the ΔΔCt formula.

### Immunohistochemistry

Paraffin embedded sections of normal and tumor breast tissue samples are collected at Northwestern Memorial Hospital. The immunohistochemistry (IHC) protocol was validated in the Northwestern Pathology Core laboratory for the FTO (SDIX, Newark, DE) and FABP4 (Epitomics, Burlingame, CA) antibodies. Sections were deparaffinized, heat (digital pressure cooker, 30 sec at 125°C) induced antigen-retrieval in citrate buffer (10 mM, pH 6.0) was used then followed by endogenous peroxidase activity blocking for 10 min at room temperature incubation in 3% hydrogen peroxide. Non-specific binding sites were blocked using Dako Protein Block (Dako, Carpinteria, CA). The FTO and FABP4 antibodies, diluted at 1:200 and 1:50 respectively, were applied on sections and incubated for 30-60 minutes at room temperature followed by a TBST (Dako, Carpinteria, CA) rinse. The secondary antibody, anti-rabbit (Dako, Carpinteria, CA) conjugated to dextran labeled polymer and horse radish peroxidase is applied for 15 min and then washed with TBST. Sections are counterstained with Mayer's Hematoxylin (Sigma, St. Louis, MO) and mounted with cover glass. All tissue samples were read by an independent pathologist (Dr Siziopikou).

### Statistical Analysis

We used logistic regression models to analyze our data with two different methods. The first method simultaneously fit all main effects of the four SNPs, referred to as multiple-SNP nonepistatic analysis. We also used the traditional logistic regression analysis based on single SNPs to confirm our findings from the multiple-SNP nonepistatic analysis. The second method was the multiple-SNP epistatic analysis, simultaneously fitting all main effects and epistatic interactions using multiple logistic regression models. The motivation for including epistatic interactions is to understand how the genetic effect(s) of a SNP varies with the genotypes at another SNP, and to aid discovery of additional genetic variants. We first performed our analyses without any covariates, and then adjusted for race, age, and BMI by including them as covariates.

We coded the main-effect predictors using the commonly-used Cockerham genetic model, and constructed all epistatic interactions by multiplying two corresponding main-effect variables. We denoted common homozygote (i.e., the homozygote with higher frequency), heterozygote, and rare homozygote for each SNP by c, h, and r, respectively. The Cockerham model defines two main effects for each SNPs (i.e., additive and dominance effects) and four epistatic interactions for two SNPs (i.e., additive-additive, additive-dominance, dominance-additive, and dominance-additive interactions)[14,15]. The additive contrast is defined as -1, 0, and 1 for c, h, and r, and the dominance contrast is defined as -0.5 for c and r and 0.5 for h, respectively. The additive effect represents the genotypic effect (r - c)/2, and the dominance effect measures h - (c + r)/2 in the logit probability of being cases. A positive additive effect (i.e., OR > 1) indicates that the rare homozygote increases cancer risk compared to the common homozygote, and a positive dominance effect means that the heterozygote increases risk compared to the mean of two homozygotes.

Multiple-SNP analyses can accommodate LD among the SNPs and have the advantages of providing potentially increased power and reduced false positives to detect causal variants, of better separating highly correlated predictors, and of more efficiently detecting epistatic interactions [16-19]. However, traditional multiple logistic regression models and procedures are highly problematic when strong LD exists and/or the number of effects is large. Thus, our multiple-SNP analyses employed Bayesian hierarchical logistic models using prior distributions for genetic effects that constrain the effects to lie in a reasonable range and results in identifiable models [20]. Specifically, we used Student-t prior distributions, i.e.,  and , with the hyperparameters (ν, *s*) reasonably preset. For main effects, we set (ν, *s*) to be (1, 2.5), which results in the routinely-used weakly informative prior [20]. For epistatic effects, we chose (1, 2.5 K_main_/K_epistasis_), where K_main _(K_epistasis_) is the total number of main (epistatic) effects in the model. This prior applies more stringent restrictions on interactions. We fitted our hierarchical logistic models by incorporating an approximate EM (expectation-maximum) algorithm into the usual iteratively weighted least squares for classical logistic regression [19].

We used two summary measures, the deviance and the Akaike information criterion (AIC), to compare different models. The deviance, defined as -2 times the log-likelihood, is a statistical summary of model fit; lower deviance means better fit to data. The AIC, defined as deviance + 2· (number of predictors), measures the predictive power; a model is estimated to reduce out-of-sample prediction error if the AIC decreases.

To evaluate the risk prediction ability of the multiple-SNP logistic models, we calculated the true positive rate (TPR, or sensitivity) and the false positive rate (FPR, or 1 - specification) that the model discriminates cases and controls. TPR (or FPR) is defined as probability that an individual is predicted as a case given that this person is truly affected (or unaffected). Because our model fitting procedure yields the predicted probabilities of a case, we could compute different sets of TPR and FPR by changing the classification threshold between 0 and 1, and thus plot the receiver operating characteristic (ROC) curves.

A linear regression model with FTO as the response and the indicator variable (for example, Tumor or Normal) as explanatory predictor was used to test the difference of the FTO tissue expression between the groups. This method is equivalent to a t test. Outliers were removed from the analysis.

## Results

### Demographics and Genotype frequencies of cases and controls

This study included 354 cases and 364 controls. As shown in Table 1 overall the median age for the cases was 50 and for the controls 47. This difference was insignificant (*p*-value = 0.6). The majority of cases and controls were Caucasian although other ethnic groups were represented as well. Cases and controls had similar ethnicity. The differences of weight and BMI among cases and controls were small but nonetheless significant. Table 1 also shows category variables for cases. Estrogen receptor (ER) and progesterone receptor (PR) status were categorized as positive or negative; tumor grade as 1, 2 or 3; stage as I, II and III/IV; Her2 status as positive or negative; Presence of lymph node metastases at diagnosis (LN) as yes or no; and family history as positive or negative. Among cases the majority were diagnosed at stage I or II (72.8%), were ER positive (78.9%) and Her2 negative (82.0%). Table 2 shows the distribution of the different alleles between cases and controls for the four *FTO *SNPs analyzed in this study. We tested for Hardy-Weinberg equilibrium (HWE) for each SNP among controls. No significant deviations from HWE were found.

**Table 1:** Baseline Characteristics of breast cases and controls

	Cases N = 354 n (%)	Controls N = 364 n (%)	*p*-value
**Age (yr): median (range)**	50 (24-83)	47 (19-87)	0.60

**Race**			
**Caucasian**	250 (72.5)	263 (72.3)	-
**Black**	56 (16.2)	59 (16.3)	0.99
**Asian**	19 (5.5)	26 (7.1)	0.34
**Hispanic**	13 (3.8)	9 (2.7)	0.40
**Other**	7 (2.0)	6 (1.6)	0.71

**Weight (lbs): median (range)**	155 (96-317)	157 (93-435)	0.03

**Height (in): median (range)**	65 (59-71)	65 (52-73)	0.66

**BMI (kg/m**^**2**^**): median (range)**	26.07 (17.61-51.43)	26.07 (16.91-79.61)	0.04

**Tumor grade**			
**1**	52 (14.7)		
**2**	132 (37.3)		
**3**	118 (33.3)		

**Stage at diagnosis**			-
**I**	106 (31.4)		
**II**	140 (41.4)		
**III/IV**	92 (27.2)		

**ER status**			-
**+**	270 (78.9)		
-	72 (21.1)		

**PR status**			-
**+**	238 (70.8)		
-	98 (29.2)		

**Her2 status**			-
**+**	58 (18.0)		
-	264 (82.0)		

**LN status**			-
**+**	176 (53.8)		
-	151 (46.2)		

**Family history**			-
**+**	138 (48.1)		
-	149 (51.9)		

**Median follow-up (mo)**	29		-

**Table 2:** Genotype characteristics of cases and controls.

SNP	Genotype	Cases N = 354 n (%)	Controls N = 364 n (%)	*p*-value
**rs9939609**	AA (c)	129 (42.7)	136 (38.9)	0.92
	AT (h)	124 (41.1)	161 (46.1)	
	TT (r)	49 (16.2)	52 (14.8)	

**rs1477196**	AA (c)	123 (39.5)	164 (45.8)	0.91
	AG (h)	130 (41.8)	154 (43.0)	
	GG (r)	58 (18.6)	40 (11.2)	

**rs7206790**	CC (c)	102 (32.4)	121 (33.8)	0.38
	CG (h)	142 (45.1)	163 (45.5)	
	GG (r)	71 (22.5)	74 (20.7)	

**rs8047395**	AA (c)	89 (28.8)	101 (28.5)	0.86
	AG (h)	140 (45.3)	172 (48.5)	
	GG (r)	80 (25.9)	82 (23.1)	

The *p*-value is for testing the deviation from Hardy-Weinberg equilibrium (HWE) among controls.

The notation c, h, and r represent common homozygote, heterozygote, and rare homozygote, respectively.

### LD Analysis

We calculated the LD measures, *D' *and *r*^*2 *^from the control samples for different races. Due to the small sample sizes of several races (Table 1), we only used Caucasian, Black, and Asian samples. To compare our LD patterns with those from the HapMap project, we extracted LD values from corresponding HapMap samples from U.S. residents with northern and western European ancestry (CEU), Ibadan of Nigeria (YRI), and Tokyo of Japan (JPT), respectively. Additional File 1 shows the *D' *and *r*^*2 *^values from our study and the HapMap project. The LD values from our study were comparable with those from the HapMap project. We found that four SNPs were in strong LD for Caucasian and Asian samples but had reduced LD values for Black samples.

### Non-epistatic analysis

We performed multiple-SNP nonepistatic analyses to simultaneously estimate the main effects of all SNPs on breast cancer risk. Table 3 shows the estimated odds ratios along with the 95% confidence intervals for all main effects under three non-epistatic models. From these analyses we found that rs1477196 was significantly associated with the risk of breast cancer through the additive effect. When taking into account race, age and BMI, the odds ratio of the additive effect of rs1477196 was 2.611 (95% CI: 1.56-4.37), indicating that compared to the common homozygote AA the rare homozygote GG significantly increased the risk of breast cancer.

**Table 3:** Multiple-SNP nonepistatic and single SNP models: odds ratios (OR), and 95% confidence intervals for all main effects of SNPs.

	Additive effects			Dominance effects		
**Nonepistatic**	**Model 1**^**a**^	**Model 2**	**Model 3**	**Model 1**	**Model 2**	**Model 3**

**rs9939609**	0.919 (0.56-1.50)	0.935 (0.56-1.55)	0.956 (0.57-1.61)	0.780 (0.52-1.17)	0.796 (0.52-1.20)	0.778 (0.50-1.12)

**rs1477196**	2.379** (1.46-3.87)	2.436** (1.48-4.00)	2.611** (1.56-4.37)	0.820 (0.55-1.22)	0.787 (0.52-1.17)	0.727 (0.47-1.10)

**rs7206790**	1.573 (0.97-2.54)	1.599 (0.98-2.61)	1.652 (0.98-2.78)	1.131 (0.76-1.68)	1.140 (0.76-1.70)	1.155 (0.76-1.76)

**rs8047395**	0.772 (0.45-1.30)	0.811 (0.47-1.38)	0.787 (0.45-1.37)	1.017 (0.66-1.55)	1.041 (0.67-1.60)	1.073 (0.68-1.69)

	**Additive effects**			**Dominance effects**		

**Single SNP**	**Model 1**^**a**^	**Model 2**	**Model 3**	**Model 1**	**Model 2**	**Model 3**

**rs9939609**	0.997 (0.79,1.25)	0.975 (0.77,1.23)	0.992 (0.78, 1.26)	0.814 (0.59,1.13)	0.831 (0.60,1.16)	0.776 (0.55,1.09)

**rs1477196**	1.390* (1.10,1.76)	1.408* (1.11,1.79)	1.447* (1.13,1.85)	0.809 (0.58,1.13)	0.781 (0.56,1.09)	0.693* (0.49,0.98)

**rs7206790**	1.066 (0.87,1.32)	1.054 (0.85,1.31)	1.022 (0.82,1.28)	0.969 (0.71,1.32)	0.980 (0.72,1.34)	0.949 (0.69,1.31)

**rs8047395**	1.052 (0.85,1.30)	1.054 (0.85,1.31)	1.075 (0.86,1.34)	0.878 (0.65,1.19)	0.876 (0.64,1.19)	0.812 (0.59.1.12)

*p-value < 0.01

**p-value < 0.001

^a ^Model 1: without covariate; Model 2: adjust for race and age; Model 3: adjust for race, age and BMI

We performed single SNP analyses to confirm the main effects of all SNPs on breast cancer risk. Table 3 shows the estimated odds ratios along with the 95% confidence intervals for all main effects under three models. From these analyses we confirmed that rs1477196 was significantly associated with the risk of breast cancer through the additive effect, indicating that compared to the common homozygote AA the rare homozygote GG significantly increased the risk of breast cancer. The *p*-values of additive effect in three models were 0.0055, 0.005, and 0.003, respectively. After the Bonferroni Correction for the multiple testing, the *p*-values were 0.044, 0.04, and 0.024, respectively, which were still significant at the nominal level of 0.05,

### Epistatic analysis

When using the multiple-SNP epistatic analysis we were able to simultaneously take into account additive and dominance effects, additive-additive, additive-dominance, dominance-additive, and dominance-dominance interactions between SNPs. Table 4 shows the estimated odds ratios and their 95% confidence intervals for all main effects under three epistatic models. Under the epistatic models, all the four FTO SNPs were significantly associated with breast cancer. Adjusted for the covariates, race, age and BMI, rs9939609 was significantly associated with the risk of breast cancer through both the additive (OR = 3.719; 95% CI: 1.43-9.68) and dominance effects (OR = 0.506; 95% CI: 0.30-0.88). Rs1477196 was also significantly associated with breast cancer through both the additive (OR = 30.111; 95% CI: 9.65-94.80) and dominance effects (OR = 0.458; 95% CI: 0.27-0.77). Finally the additive effects of rs7206790 (OR = 1.888; 95% CI: 1.08-3.29) and rs8047395 (OR = 0.281; 95% CI: 0.12-0.68) were significantly associated with breast cancer.

**Table 4:** Epistatic models: odds ratios (OR) and 95% confidence intervals (95% CI) for all main effects of SNPs.

	Additive effects			Dominance effects		
	^**a**^**Model 1**	**Model 2**	**Model 3**	**Model 1**	**Model 2**	**Model 3**

**rs9939609**	3.515* (1.41-8.708)	3.281* (1.32-8.16)	3.719* (1.43-9.68)	0.515* (0.31-0.84)	0.531* (0.32-0.87)	0.506* (0.30-0.88)

**rs1477196**	31.589** (10.27-97.11)	27.551** (8.98-84.52)	30.111** (9.65-94.80)	0.509* (0.31-0.83)	0.515* (0.32-0.84)	0.458* (0.27-0.77)

**rs7206790**	1.865* (1.11-3.13)	1.802* (1.07-3.03)	1.888* (1.08-3.29)	1.074 (0.71-1.63)	1.094 (0.72-1.66)	1.088 (0.68-1.65)

**rs8047395**	0.247* (0.10-0.61)	0.262* (0.11-0.64)	0.281* (0.12-0.68)	0.822 (0.52-1.30)	0.841 (0.53-1.33)	0.847 (0.52-1.38)

*p-value < 0.05, **p-value < 0.001

^a^Model 1: without covariate; Model 2: adjust for race and age; Model 3: adjust for race, age and BMI

Table 5 shows significant epistatic effects with *p*-value < 0.05 under three models. All the three analyses detected two significant epistatic interactions, additive-additive interaction between rs1477196 and rs9939609 and dominance-dominance interaction between rs1477196 and rs8047395. When taking into account race, age and BMI, the estimated odds ratios of these two interactions were 12.031 (95%CI: 2.68-53.95) and 0.054 (95%CI: 0.01-0.20).

**Table 5:** Epistatic analyses: odds ratios (OR) and 95% confidence intervals (95% CI) for epistatic effects with *p*-value < 0.05.

Epistatic effect	^**a**^**Model 1**	Model 2	Model 3
**rs1477196a:rs9939609a**	11.525** (2.76-48.17)	9.773* (2.32-41.21)	12.031* (2.68-53.95)

**rs1477196d:rs8047395d**	0.0416** (0.01-0.15)	0.048** (0.01-0.18)	0.054** (0.01-0.20)

The term X1:X2 represents interaction between X1 and X2.

*p-value < 0.05, **p-value < 0.001

^a^Model 1: without covariate; Model 2: adjust for race and age; Model 3: adjust for race, age and BMI

Based on the fitted epistatic models, we calculated the odds ratios of three genotypes for each SNP and the probabilities of being case for two SNP pairs, rs1477196 and rs9939609, rs1477196 and rs8047395, which showed significant interactions. Additional File 2 and 3 display the results from the epistatic models including race, age, and BMI as covariates. Other epistatic models produced similar results. From Additional File 2, we can see that the rare homozygotes of rs9939609, rs1477196, and rs7206790 increased the risk of breast cancer compared to the common homozygotes, while the common homozygote for rs8047395 was associated with an increased risk. Additional File 3 shows that the genetic effect(s) of a SNP varied with the genotypes at another SNP.

### Model comparison

We estimated deviance and AIC in the multiple-SNP nonepistatic and epistatic analyses under the three models (Additional File 4). The epistatic models had lower deviance and AIC than the non-epistatic models. This indicated that inclusion of the significant epistatic interactions improved the fit of the model to data and reduced out-of-sample prediction error. The epistatic model adjusting for age, race and BMI had the lowest deviance and AIC and thus best fit to data. For this reason we have selected this model for the interpretation of our results.

### Breast cancer risk prediction

To evaluate the ability of the multiple-SNP models of the *FTO *pathway genotypes to predict breast cancer risk we plotted the receiver operating characteristics (ROC) curve (Additional File 4). The ROC curve shows the estimated true positive and the false positive rates for different classification thresholds between 0 and 1. The area under the ROC curve (AUC) was used to measure the discrimination power of our genotypes. The AUC number represents the probability that given two random individuals, one who will develop the disease and the other who will not, the genotypes will assign the former as a positive test and the latter as a negative test. A perfect genotype would have an AUC of 1. It has been suggested that an AUC > 0.5 has some discriminatory ability and for screening high risk individuals an AUC > 0.75 should be used[21].

We performed analyses based on three models: the epistatic model adjusted for race, age and BMI, the nonepistatic model adjusted for race, age and BMI, and the model including race, age and BMI and no SNPs. The epistatic model achieved higher true positive rate and lower false positive rate than the nonepistatic model did for any thresholds. The areas under the ROC curves (AUC) were 0.68, 0.60, and 0.53, respectively. This indicated that *FTO *genotypes provided powerful classifiers to predict breast cancer risk and a model with epistatic interactions further improved the prediction accuracy.

### Subgroup analyses

We estimated category-specific risks by comparing the genotype distribution of cases and controls within each category for age or between each case category and all controls for case category variables, i.e., tumor grade, stage at diagnosis, ER, PR, Her2, LN, and family history (see Table 1). To investigate the effects of age, subjects were separated into four groups (under 39, 39-49, 50-59 and 60+) according to age at diagnosis (cases) or interview (controls). Since sample sizes were largely reduced, all these subgroup analyses fit only main effects.

Table 6 displays the estimated odd ratios and their 95% confidence intervals for significant effects (i.e., *p*-value < 0.05). These analyses showed that rs1477196 is associated with breast cancer regardless of ER or PR status, grade of diagnosis, presence or absence of axillary lymph nodes or family history. Rs1477196 was also associated with stage II, III and IV at diagnosis although it was not significantly associated with stage I breast cancer. Other subset analyses showed that rs7206790 was mostly associated with ER and PR+ disease, stage III/IV, grade II, presence of axillary LN and positive family history. Finally rs8047395 was associated with grade III breast cancer.

**Table 6:** Subgroup analyses: odds ratios (OR) and 95% confidence intervals (95% CI) for main effects with *p*-value < 0.05.

	Age < 39	ER-	ER+		PR-	PR+
	**Additive**	**Additive**	**Additive**		**Additive**	**Additive**

**rs1477196**	5.209* (1.61-16.91)	2.466* (1.05-5.79)	2.437** (1.44-4.11)		2.202* (1.05-4.64)	2.649** (1.54-4.57)

**rs7206790**	3.487* (1.22-9.97)		1.81* (1.08-3.02)			1.860* (1.08-3.21)

**rs8047395**		0.349* (0.14-0.89)				

	**Stage II**	**Stage III/IV**	**Grade I**		**Grade II**	**Grade III**

	**Additive**	**Additive**	**Additive**	**Dominance**	**Additive**	**Additive**

**rs1477196**	2.368* (1.21-4.63)	3.640* (1.63-8.14)	2.766* (1.07-7.13)	0.345* (0.15-0.78)	2.405* (1.22-4.75)	3.067* (1.49-6.31)

**rs7206790**		2.319* (1.12-4.79)			1.921* (0.99-3.71)	

**rs8047395**						0.361* (0.16-0.78)

	**No LN**	**Yes LN**	**Her2-**		**Family hx -**	**Family hx +**

	**Additive**	**Additive**	**Additive**		**Additive**	**Additive**

**rs1477196**	2.722* (1.42-5.22)	2.318* (1.28-4.18)	2.709** (1.60-4.58)		3.017** (1.60-5.67)	2.501* (1.26-4.93)

**rs7206790**		1.868* (1.04-3.35)				2.449* (1.28-4.69)

*p < 0.05, **p < 0.001

### Tissue FTO expression

When looking at FTO expression between normal and tumor tissue we found that the FTO in tumor tissue was significantly lower than in normal tissue (*p*-value < 0.001) (Additional File 5, Figure 1). This difference was similar regardless of ER, PR and Her2 expression. However there didn't seem to be any significant difference in tumor FTO expression in ER + vs ER-, PR+ vs PR- or Her2+ vs Her2- cancers (Additional File 6).

**Figure 1:**
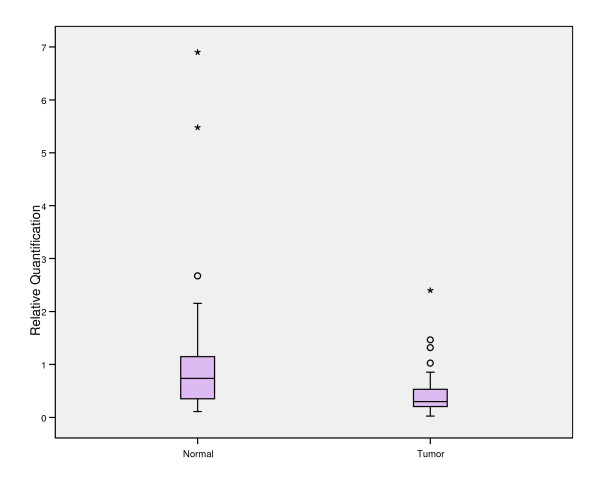
Tissue expression of FTO in normal and tumor breast tissue. o = outliers, * = extreme cases.

**Figure 2:**
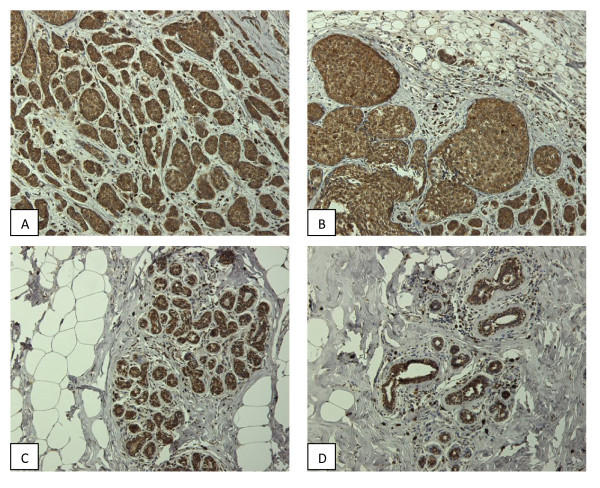
**Breast cancer (A,B) and normal breast tissue (C,D) staining of FTO**. As shown here there is strong cytoplasmic staining for FTO in the breast tissue whereas the surrounding adipose tissue and stroma don't show any staining for FTO.

To evaluate whether FTO was expressed in the breast tissue or surrounding area we performed IHC staining on 21 breast cancer tumors and 20 samples of normal breast epithelium. We found that all samples both malignant and normal breast epithelium showed positive cytoplasmic staining for FTO (Figure 2). We could not detect any differences between staining intensity between normal and malignant breast tissue. Adipose tissue had little or no staining of FTO. We also performed staining for FABP4, an adipocyte marker, and found that seven of 18 (38.8%) breast tumors stained positive and three of 20 (15%) normal breast epithelium stained positive, whereas all adipose tissue stained positive for FABP4.

## Discussion

In this clinic-based case-control study we found that SNPs located in intron 1 of *FTO *are associated with breast cancer risk. More specifically rs1477196, rs9939609, rs7206790 and rs8047395 were significantly associated with breast cancer risk. Furthermore, with an AUC of 0.68 these *FTO *genotypes provided powerful classifiers to predict breast cancer risk. This is to our knowledge the first report of an association between *FTO *and breast cancer risk.

There has also been extensive research on the association of DM and breast cancer. In a recent meta-analysis of 20 case-control studies there was a 20% increased risk for breast cancer in women with DM[6]. In the four cohort studies, which were included in the same meta-analysis, breast cancer risk increased by 24% in patients with DM. We recently showed that adiponectin, an adipokine which is linked to obesity and type II DM, plays an important role in breast cancer risk[22-24]. We therefore sought out to find other links between obesity, DM and breast cancer, which lead us to evaluate the role of *FTO *in breast cancer risk.

We performed several analyses using epistatic and nonepistatic genetic models. The nonepistatic models take into account all SNPs simultaneously (Multiple-SNP analysis). In the epistatic models the interaction between the SNPs is also taken into account as to how it changes individual SNP effects. In the nonepistatic model rs1477196 was found to be significantly associated with breast cancer risk. Of all models performed the epistatic model, which controls for age, race and BMI had the best fit. That model showed that all four SNPs (rs1477196, rs9939609, rs7206790 and rs8047395) are significantly and jointly associated with breast cancer risk. Furthermore, the interactions between rs1477196 and rs9939609, as well as, rs1477196 and rs8047395 were significantly associated with breast cancer risk.

In subset analyses we observed that rs1477196 was significantly associated with breast cancer regardless of hormone receptor status, tumor grade, stage at diagnosis or presence of family history. However rs7206790 predicted hormone receptor positive breast cancers, in young women with a positive family history and was associated with more advanced stages at diagnosis.

We performed a breast cancer risk prediction model using the ROC curve. This curve utilizes the genotypes measured in all individuals and assesses the combination of sensitivity and specificity of different combinations of genotypes. We observed an AUC of 0.68 for the epistatic model, significantly higher than the models ignoring epistasis and genetic factors. This result shows that there is utility of prediction of breast cancer risk using the *FTO *genotype. It has been suggested by others that an AUC > 0.5 has some discriminatory ability [21]. In a recent paper by Watcholder et al[25] et al a genetic model, that included 10 SNPs found in genome wide association studies (GWAS) to be associated with breast cancer risk, produced an AUC of 58.9%. In that study the SNP-only model predicted risk slightly better than the Gail model. Another study by Mealiffe et al[26] also confirmed that a SNP-only model produced a higher AUC (58.7%) compared with the Gail model (55.7%). The AUCs in these studies are somewhat lower compared with the AUC obtained in our study. The major reasons for this prediction improvement are: 1) we used a Bayesian hierarchical model to fit the data and to predict the disease risk. As described in Gelman et al. [27], a Bayesian hierarchical model can improve the prediction accuracy; 2) the previous studies have not included interactions into the predictive models. If genetic interactions are present, adding these interactions to a predictive model can increase the accuracy of prediction[28-30]. However we do recognize that this is a single study and we would need to validate our results before we can be certain of the magnitude of our findings.

We also showed the presence of FTO in breast tissue and found that normal breast tissue had significantly higher FTO RNA expression compared with breast cancer tissue. Furthermore, when comparing subtypes of breast cancer risk by ER, PR and Her2 expression we did not find any significant differences in FTO RNA expression according to these subtypes. We confirmed the presence of FTO expression by performing IHC where we found that both normal breast tissue and breast cancer tissue have a positive cytoplasmic stain for FTO. FTO has a potential role in nucleic acid demethylation. Its expression in breast tissue points toward an important role in the demethylation process[12]. Recent studies have shown that mice overexpressing *FTO *are obese. Moreover, ubiquitous overexpression of *FTO *has been shown to lead to a dose-dependent increase in body and fat mass, irrespective of whether mice are fed a standard or a high-fat diet suggesting that increased body mass results primarily from increased food intake[31].

Our study has several strengths and limitations. Cases and controls were similar in respect to age, race and geographic location but differed significantly in regard to BMI. However we controlled for age, race and BMI. Information on chronic diseases was not recorded for cases and controls with the possibility of discrepancy between the two groups. However FTO has only shown to play a role in obesity and we controlled for BMI. Therefore such discrepancy would likely not affect our findings. The selection of our SNPs was based on previous data from GWAS that correlated them with obesity. Although exposure was assessed in the context of a case control study, it is impossible that breast cancer would have changed SNP classification, which is already determined at birth. Thus this study does fulfill the "time sequence" criterion for causality. This in association with existing epidemiologic evidence and biologic plausibility support a causal association between these SNPs and risk for breast cancer. Strengths of the study include the use of state of the art methods for evaluation of SNPs as well as FTO IHC and that we have controlled for known potential confounders. Confounding from unknown factors remains a possibility, however. Some random misclassification is unavoidable but such misclassification leads to suppression of effect estimates and p-values away from statistical significance. Thus, our statistically significant results of this study could not have been attributed to misclassification.

## Conclusions

To our knowledge this is the first study reporting an association between FTO pathway SNPs and breast cancer risk. This is also the first study to show expression of FTO in normal and malignant breast tissue. If confirmed in subsequent studies, our findings suggest that genetic variants of *FTO *alter breast cancer risk. The fact that FTO is expressed in human breast tissue as well as its role in nucleic acid demethylation[12] point toward a direct effect of FTO in breast cancer. The FTO axis may emerge as an important modifier of breast cancer risk. Our findings point toward an important role of FTO in breast cancer.

## Abbreviations

FTO: Fat mass and obesity gene; SNP: single nucleotide polymorphism; AIC: Akaike information criterion; GWAS: genome wide association study; ROC: Receiver operating characteristic; AUC: area under the curve; DM: diabetes mellitus; IRB: institutional board review; LD: Linkage disequilibrium; RT-PCR: real time polymerase chain reaction; ER: estrogen receptor; PR: progesterone receptor; LN: lymph node; Her2: human epidermal receptor 2; DNA: Deoxyribonucleic acid; IHC: immunohistochemistry; OR: Odds ratio; CI: confidence interval; TPR: true positive rate; FPR: false positive rate; FABP4: fatty acid binding protein 4.

## Competing interests

The authors declare that they have no competing interests.

## Authors' contributions

VK participated in writing the paper and design of the study, NY participated in the statistical analysis of the paper, MS participated in genotyping and expression assays of the study, KS participated in the immunohistochemistry and the interpretation of the study, KZ participated in the statistical analysis of the paper, YX participated in the expression assays of the study, ST participated in the recruitment of cases and controls, SA participated in the recruitment of cases and controls, BP participated in the conception of the study and recruitment of cases and controls, CM participated in writing the paper and conception of the study. All authors read and approved the final manuscript.

## Acknowledgements

This work was supported by the Walter S. Mander Foundation, Chicago, IL, the Lynn Sage Foundation, Chicago, IL, the Lynn Sage Scholar Award, Chicago, IL (Kaklamani); R01 CA112520 (Pasche), R01-GM74913 (Zhang) and 2R01GM069430-06 (Yi). In loving memory of Dolores Knes, who reminds us every day that the goal of our research is to improve the lives of our patients.

## Pre-publication history

The pre-publication history for this paper can be accessed here:

http://www.biomedcentral.com/1471-2350/12/52/prepub

## Supplementary Material

Additional file 1 **LD analysis for SNPs**. Odds ratios (ORs) of three genotypes for the four SNPs under the epistatic model adjusted for race, age and BMI. The notation c, h, and r represent common homozygote, heterozygote, and rare homozygote, respectively. Click here for file

Additional file 2 **Odds ratios (ORs) of three genotypes for the four SNPs under the epistatic model adjusted for race, age and BMI**. The notation c, h, and r represent common homozygote, heterozygote, and rare homozygote, respectively. Click here for file

Additional file 3 **Probablities of being case for two-locus genotypes at SNPs that show significant interactions under the epistatic model adjusted for race, age and BMI**. The notation c, h, and r represent common homozygote, heterozygote, and rare homozygote, respectively. Click here for file

Additional file 4 **Receiver operating characteristic (ROC) curves for risk prediction for 1**. epistatic model adjusted for race, age and BMI (dotted black line), 2. nonepistatic model adjusted for race, age and BMI (solid black line), and 3. model with covariates, race, age and BMI, and no SNPs. The areas under the ROC curves (AUC) are 0.68, 0.60, and 0.53, respectively. Click here for file

Additional file 5 **Results for FTO tissue expression**. Results for FTO tissue expression. Click here for file

Additional file 6 **FTO expression according to ER, PR and her2 status**. Tissue expression of FTO. TERneg: tumor ER negative, TERpos: tumor ER positive, TPRneg: tumor PR negative; TPRpos: tumor PR positive, THer2neg: Tumor Her2 negative, THer2pos: tumor Her2 positive. o = outliers, * = extreme cases. Click here for file

## References

[B1] Jemal A Siegel R Ward E Hao Y Xu J Thun MJ Cancer statistics, 2009 CA Cancer J Clin 2009 59 225 249 10.3322/caac.20006 19474385

[B2] Wolk A Gridley G Svensson M Nyren O McLaughlin JK Fraumeni JF A prospective study of obesity and cancer risk (Sweden) Cancer Causes Control 2001 12 13 21 10.1023/A:1008995217664 11227921

[B3] Huang Z Hankinson SE Colditz GA Stampfer MJ Hunter DJ Manson JE Dual effects of weight and weight gain on breast cancer risk JAMA 1997 278 1407 1411 10.1001/jama.278.17.1407 9355998

[B4] Harvie M Howell A Vierkant RA Kumar N Cerhan JR Kelemen LE Association of gain and loss of weight before and after menopause with risk of postmenopausal breast cancer in the Iowa women's health study Cancer Epidemiol Biomarkers Prev 2005 14 656 661 10.1158/1055-9965.EPI-04-0001 15767346

[B5] Prentice RL Caan B Chlebowski RT Patterson R Kuller LH Ockene JK Low-fat dietary pattern and risk of invasive breast cancer: the Women's Health Initiative Randomized Controlled Dietary Modification Trial JAMA 2006 295 629 642 10.1001/jama.295.6.629 16467232

[B6] Larsson SC Mantzoros CS Wolk A Diabetes mellitus and risk of breast cancer: a meta-analysis Int J Cancer 2007 121 856 862 10.1002/ijc.22717 17397032

[B7] Cho YM Kim TH Lim S Choi SH Shin HD Lee HK Type 2 diabetes-associated genetic variants discovered in the recent genome-wide association studies are related to gestational diabetes mellitus in the Korean population Diabetologia 2009 52 253 261 10.1007/s00125-008-1196-4 19002430

[B8] Grant SF Li M Bradfield JP Kim CE Annaiah K Santa E Association analysis of the FTO gene with obesity in children of Caucasian and African ancestry reveals a common tagging SNP PLoS One 2008 3 e1746 10.1371/journal.pone.0001746 18335027 PMC2262153

[B9] Thorleifsson G Walters GB Gudbjartsson DF Steinthorsdottir V Sulem P Helgadottir A Genome-wide association yields new sequence variants at seven loci that associate with measures of obesity Nat Genet 2009 41 18 24 10.1038/ng.274 19079260

[B10] van HM Dehghan A Witteman JC van Duijn CM Uitterlinden AG Oostra BA Predicting type 2 diabetes based on polymorphisms from genome-wide association studies: a population-based study Diabetes 2008 57 3122 3128 10.2337/db08-0425 18694974 PMC2570410

[B11] Zhao J Bradfield JP Li M Wang K Zhang H Kim CE The Role of Obesity-associated Loci Identified in Genome-wide Association Studies in the Determination of Pediatric BMI Obesity (Silver Spring) 2009 19478790 10.1038/oby.2009.159 PMC2860782

[B12] Gerken T Girard CA Tung YC Webby CJ Saudek V Hewitson KS The obesity-associated FTO gene encodes a 2-oxoglutarate-dependent nucleic acid demethylase Science 2007 318 1469 1472 10.1126/science.1151710 17991826 PMC2668859

[B13] Hinney A Nguyen TT Scherag A Friedel S Bronner G Muller TD Genome wide association (GWA) study for early onset extreme obesity supports the role of fat mass and obesity associated gene (FTO) variants PLoS One 2007 2 e1361 10.1371/journal.pone.0001361 18159244 PMC2137937

[B14] Cordell HJ Epistasis: what it means, what it doesn't mean, and statistical methods to detect it in humans Hum Mol Genet 2002 11 2463 2468 10.1093/hmg/11.20.2463 12351582

[B15] Zeng ZB Wang T Zou W Modeling quantitative trait Loci and interpretation of models Genetics 2005 169 1711 1725 10.1534/genetics.104.035857 15654105 PMC1449562

[B16] Balding DJ A tutorial on statistical methods for population association studies Nat Rev Genet 2006 7 781 791 10.1038/nrg1916 16983374

[B17] Hoggart CJ Whittaker JC De Iorio M Balding DJ Simultaneous analysis of all SNPs in genome-wide and re-sequencing association studies PLoS Genet 2008 4 e1000130 10.1371/journal.pgen.1000130 18654633 PMC2464715

[B18] Malo N Libiger O Schork NJ Accommodating linkage disequilibrium in genetic-association analyses via ridge regression Am J Hum Genet 2008 82 375 385 10.1016/j.ajhg.2007.10.012 18252218 PMC2427310

[B19] Yi N Banerjee S Hierarchical generalized linear models for multiple quantitative trait locus mapping Genetics 2009 181 1101 1113 10.1534/genetics.108.099556 19139143 PMC2651046

[B20] Yi N Kaklamani VG Pasche B Bayesian analysis of genetic interactions in case-control studies, with application to adiponectin genes and colorectal cancer risk Ann Hum Genet 2011 75 90 104 10.1111/j.1469-1809.2010.00605.x 20846215 PMC3005151

[B21] Janssens AC Moonesinghe R Yang Q Steyerberg EW van Duijn CM Khoury MJ The impact of genotype frequencies on the clinical validity of genomic profiling for predicting common chronic diseases Genet Med 2007 9 528 535 10.1097/GIM.0b013e31812eece0 17700391

[B22] Kaklamani VG Sadim M Hsi A Offit K Oddoux C Ostrer H Variants of the adiponectin and adiponectin receptor 1 genes and breast cancer risk Cancer Res 2008 68 3178 3184 10.1158/0008-5472.CAN-08-0533 18451143 PMC2685173

[B23] Mantzoros C Petridou E Dessypris N Chavelas C Dalamaga M Alexe DM Adiponectin and breast cancer risk J Clin Endocrinol Metab 2004 89 1102 1107 10.1210/jc.2003-031804 15001594

[B24] Mantzoros CS Li T Manson JE Meigs JB Hu FB Circulating adiponectin levels are associated with better glycemic control, more favorable lipid profile, and reduced inflammation in women with type 2 diabetes J Clin Endocrinol Metab 2005 90 4542 4548 10.1210/jc.2005-0372 15914524

[B25] Wacholder S Hartge P Prentice R Garcia-Closas M Feigelson HS Diver WR Performance of common genetic variants in breast-cancer risk models N Engl J Med 2010 362 986 993 10.1056/NEJMoa0907727 20237344 PMC2921181

[B26] Mealiffe ME Stokowski RP Rhees BK Prentice RL Pettinger M Hinds DA Assessment of clinical validity of a breast cancer risk model combining genetic and clinical information J Natl Cancer Inst 2010 102 1618 1627 10.1093/jnci/djq388 20956782 PMC2970578

[B27] Gelman A Jakulin A Pittau MG Su YS A weakly informative default prior distribution for logistic and other regression models Annals of Applied Statistics 2008 2 1360 1383 10.1214/08-AOAS191

[B28] Clark AG Clegg M, et al Limits to prediction of phenotype from knowledge of genotypes. Limits to knowledge in evolutionary genetics 2000 Kluwer Academic/Penum Publishers, New York 205 224

[B29] Moore JH Williams SM Epistasis and its implications for personal genetics Am J Hum Genet 2009 85 309 320 10.1016/j.ajhg.2009.08.006 19733727 PMC2771593

[B30] Yi N Statistical analysis of genetic interactions Genetics Research 2011 in press Ref Type 10.1017/S0016672310000595 PMC3203544 21429274

[B31] Church C Moir L McMurray F Girard C Banks GT Teboul L Overexpression of Fto leads to increased food intake and results in obesity Nat Genet 2010 42 1086 1092 10.1038/ng.713 21076408 PMC3018646

