# Supplementary material for: The role of the fat mass and obesity associated gene (FTO) in breast cancer risk
Source: BMC Med Genet. 2011 Apr 13;12:52. doi: 10.1186/1471-2350-12-52 (PMC3089782; doi:10.1186/1471-2350-12-52)
Supplement: Additional file 1 — LD analysis for SNPs. Odds ratios (ORs) of three genotypes for the four SNPs under the epistatic model adjusted for race, age and BMI. The notation c, h, and r represent common homozygote, heterozygote, and rare homozygote, respectively. [file 1471-2350-12-52-S1.DOC]

**Additional File 1**. LD analysis for SNPs. Upper diagonal: LD from our data; Lower diagonal: LD from HapMap. LD was calculated from the control samples only. Due to the small sample size of several populations, only Caucasian, Black, and Asian samples were used in the calculation. Correspondingly, the CEU, YRI, and JPT samples from HapMap were used to extract the LD values.

| Race: Caucasian vs. HapMap CEU | | | | | | | | |
| --- | --- | --- | --- | --- | --- | --- | --- | --- |
|  | D’ | | | | r^2^ | | | |
|  | rs9939609 | rs1477196 | rs7206790 | rs8047395 | rs9939609 | rs1477196 | rs7206790 | rs8047395 |
| rs9939609 | NA | 1.000 | 0.894 | 0.989 | NA | 0.387 | 0.619 | 0.618 |
| rs1477196 | 1 | NA | 0.915 | 1.000 | 0.356 | NA | 0.418 | 0.612 |
| rs7206790 | 0.847 | 0.93 | NA | 0.926 | 0.536 | 0.424 | NA | 0.700 |
| rs8047395 | 1 | 1 | 0.91 | NA | 0.623 | 0.572 | 0.68 | NA |
| Race: Black vs. HapMap YRI | | | | | | | | |
|  | D’ | | | | r^2^ | | | |
|  | rs9939609 | rs1477196 | rs7206790 | rs8047395 | rs9939609 | rs1477196 | rs7206790 | rs8047395 |
| rs9939609 | NA | 0.999 | 0.388 | 0.456 | NA | 0.124 | 0.101 | 0.096 |
| rs1477196 | 1.000 | NA | 0.999 | 0.999 | 0.041 | NA | 0.185 | 0.270 |
| rs7206790 | 0.216 | 1 | NA | 0.235 | 0.033 | 0.057 | NA | 0.038 |
| rs8047395 | 0.514 | 1 | 0.396 | NA | 0.083 | 0.128 | 0.030 | NA |
| Race: Asian vs. HapMap JPT | | | | | | | | |
|  | D’ | | | | r^2^ | | | |
|  | rs9939609 | rs1477196 | rs7206790 | rs8047395 | rs9939609 | rs1477196 | rs7206790 | rs8047395 |
| rs9939609 | NA | 0.998 | 0.874 | 0.999 | NA | 0.083 | 0.546 | 0.225 |
| rs1477196 | 1 | NA | 0.817 | 1.000 | 0.073 | NA | 0.040 | 0.369 |
| rs7206790 | 0.711 | 0.628 | NA | 0.999 | 0.462 | 0.032 | NA | 0.161 |
| rs8047395 | 1.000 | 0.934 | 1.000 | NA | 0.092 | 0.698 | 0.118 | NA |
