# Supplementary material for: The role of the fat mass and obesity associated gene (FTO) in breast cancer risk
Source: BMC Med Genet. 2011 Apr 13;12:52. doi: 10.1186/1471-2350-12-52 (PMC3089782; doi:10.1186/1471-2350-12-52)
Supplement: Additional file 2 — Odds ratios (ORs) of three genotypes for the four SNPs under the epistatic model adjusted for race, age and BMI. The notation c, h, and r represent common homozygote, heterozygote, and rare homozygote, respectively. [file 1471-2350-12-52-S2.DOC]

**Additional File 2. Odds ratios (ORs) of three genotypes for the four SNPs under the epistatic model adjusted for race, age and BMI. The notation c, h, and r represent common homozygote, heterozygote, and rare homozygote, respectively.**
