# Supplementary material for: The role of the fat mass and obesity associated gene (FTO) in breast cancer risk
Source: BMC Med Genet. 2011 Apr 13;12:52. doi: 10.1186/1471-2350-12-52 (PMC3089782; doi:10.1186/1471-2350-12-52)
Supplement: Additional file 3 — Probablities of being case for two-locus genotypes at SNPs that show significant interactions under the epistatic model adjusted for race, age and BMI. The notation c, h, and r represent common homozygote, heterozygote, and rare homozygote, respectively. [file 1471-2350-12-52-S3.DOC]

**Additional File 3. Probablities of being case for two-locus genotypes at SNPs that show significant interactions under the epistatic model adjusted for race, age and BMI. The notation c, h, and r represent common homozygote, heterozygote, and rare homozygote, respectively.**
