# Supplementary material for: The role of the fat mass and obesity associated gene (FTO) in breast cancer risk
Source: BMC Med Genet. 2011 Apr 13;12:52. doi: 10.1186/1471-2350-12-52 (PMC3089782; doi:10.1186/1471-2350-12-52)
Supplement: Additional file 4 — Receiver operating characteristic (ROC) curves for risk prediction for 1. epistatic model adjusted for race, age and BMI (dotted black line), 2. nonepistatic model adjusted for race, age and BMI (solid black line), and 3. model with covariates, race, age and BMI, and no SNPs. The areas under the ROC curves (AUC) are 0.68, 0.60, and 0.53, respectively. [file 1471-2350-12-52-S4.DOC]

**Additional File 4. Receiver operating characteristic (ROC) curves for risk prediction for 1. epistatic model adjusted for race, age and BMI (dotted black line), 2. nonepistatic model adjusted for race, age and BMI (solid black line), and 3. model with covariates, race, age and BMI, and no SNPs. The areas under the ROC curves (AUC) are 0.68, 0.60, and 0.53, respectively.**
