# Supplementary material for: The role of the fat mass and obesity associated gene (FTO) in breast cancer risk
Source: BMC Med Genet. 2011 Apr 13;12:52. doi: 10.1186/1471-2350-12-52 (PMC3089782; doi:10.1186/1471-2350-12-52)
Supplement: Additional file 5 — Results for FTO tissue expression. Results for FTO tissue expression. [file 1471-2350-12-52-S5.DOC]

**Additional File 5. Results for FTO tissue expression**

|  | Efect Estimate | P value |
| --- | --- | --- |
| Tumor vs Normal | -0.385 | <0.001 |
| Tumor ER- vs Tumor ER+ | -0.061 | 0.344 |
| Tumor PR- vs Tumor PR+ | -0.087 | 0.186 |
| Tumor Her2- vs Tumor Her2+ | -0.081 | 0.383 |
