# Supplementary material for: The role of the fat mass and obesity associated gene (FTO) in breast cancer risk
Source: BMC Med Genet. 2011 Apr 13;12:52. doi: 10.1186/1471-2350-12-52 (PMC3089782; doi:10.1186/1471-2350-12-52)
Supplement: Additional file 6 — FTO expression according to ER, PR and her2 status. Tissue expression of FTO. TERneg: tumor ER negative, TERpos: tumor ER positive, TPRneg: tumor PR negative; TPRpos: tumor PR positive, THer2neg: Tumor Her2 negative, THer2pos: tumor Her2 positive. o = outliers, * = extreme cases. [file 1471-2350-12-52-S6.DOC]

**Additional File 6.** **Tissue expression of FTO. TERneg: tumor ER negative, TERpos: tumor ER positive, TPRneg: tumor PR negative; TPRpos: tumor PR positive, THer2neg: Tumor Her2 negative, THer2pos: tumor Her2 positive. o=outliers, *= extreme cases.**
